# Dynamic Deterioration Pattern of Soybean Meal Contaminated by *Fusarium graminearum*

**DOI:** 10.3390/foods15173009

**Published:** 2026-08-26

**Authors:** Miao Yu, Liangchen Zhang, Shuyuan Xing, Mingyu Wu

**Affiliations:** 1 Institute of Food and Processing, Liaoning Academy of Agricultural Sciences, Shenyang 110161, China; jannytiti@163.com (M.Y.);; 2 Food and Processing Research Institute, Shenyang Agricultural University, Shenyang 110866, China

**Keywords:** soybean meal, *Fusarium graminearum*, protein secondary structure, microstructure, volatile organic compounds

## Abstract

As a major contaminant fungus in grains and by-products, *Fusarium graminearum* rapidly colonizes and proliferates, posing safety risks to feed commodities. In this study, artificial inoculation was adopted to simulate *F. graminearum* contamination in soybean meal. Dynamic changes in fungal population, protein secondary structure, microstructure and volatile organic compounds (VOCs) were systematically monitored across a 54-day storage period. Soybean meal exhibited a three-phase deterioration pattern: latent infection (0–30 d), accelerated spoilage at day 36, and severe deterioration (42–54 d). Fungal conidia counts increased sharply before declining moderately with extended incubation. Ordered protein conformations (α-helix and β-sheet) underwent continuous degradation and converted into disordered β-turn and random coil structures, accompanied by gradual disruption of the compact microstructure of soybean meal. In total, 99 VOCs spanning 13 chemical classes were identified throughout the contamination timeline. Combining orthogonal partial least squares discriminant analysis (OPLS-DA) and Pearson correlation analysis, five volatile biomarkers tightly associated with *F. graminearum* spoilage were screened: four upregulated fungal metabolites and one downregulated endogenous flavor compound. This work characterizes the dynamic deterioration profiles and volatile fingerprint of *F. graminearum*-inoculated soybean meal under controlled laboratory conditions, delivering preliminary laboratory evidence to support the future development of potential spoilage monitoring approaches.

## 1. Introduction

Soybean meal, a major by-product of soybean oil desolventization, contains abundant, well-balanced amino acids and is a superior plant protein source for human consumption and animal feed worldwide [1,2]. Its porous structure and high water-holding capacity render soybean meal susceptible to fungal spoilage under ambient high-humidity storage. Fungal growth degrades proteins, lipids and other nutrients, impairing the processing performance and nutritional value of soybean meal. More critically, mold metabolism produces toxic mycotoxins, leading to severe economic losses and food safety risks. The Food and Agriculture Organization (FAO) estimates that mycotoxins contaminate around 25% of global agricultural crops, and 2% of grain supplies become unfit for utilization due to excessive mycotoxin concentrations [3]. Accordingly, investigating multi-dimensional quality deterioration of soybean meal across its full spoilage cycle is essential to develop early-warning and targeted control strategies for storage-associated fungal contamination.

*Fusarium graminearum* (*F. graminearum*) ranks among the most destructive fungi colonizing grains and oilseed residues [4]. Widespread in both field and warehouse environments, this pathogen exhibits strong infectivity, vigorous metabolic activity and robust tolerance to fluctuating storage and shipping conditions [5]. Previous evidence confirms that *F. graminearum* secretes extracellular hydrolases (proteases, lipases, cellulases) that disrupt the microstructure and macromolecular components of grain substrates. Meanwhile, the fungus synthesizes abundant strain-specific volatile metabolites [6] and hazardous mycotoxins, such as trichothecenes, fumonisins, moniliformin, and beauvericin [7], which are the primary drivers of soybean meal quality degradation and safety hazards.

Existing research regarding soybean meal spoilage largely concentrates on natural mixed fungal communities and the independent effects of temperature and humidity on mold severity [8]. Few studies have tracked full-cycle dynamic changes in soybean meal inoculated solely with exogenous *F. graminearum*, and no integrated analysis links fungal proliferation, protein conformational shifts, microstructural breakdown and volatile metabolic profiles. In recent years, multi-platform analytical workflows combining microscopic imaging, vibrational spectroscopy, gas chromatography–mass spectrometry and chemometrics have become standard for exploring spoilage mechanisms of cereal and oilseed commodities [9,10]. Fungal metabolism triggers protein oxidation, lipid peroxidation and polysaccharide decomposition, alongside the release of unique volatile organic compounds (VOCs) that serve as potential spoilage biomarkers. Specifically, Fourier-transform infrared spectroscopy (FTIR) quantifies alterations in protein secondary structures, scanning electron microscopy (SEM) visualizes tissue damage, and headspace solid-phase microextraction coupled with gas chromatography–mass spectrometry (HS-SPME-GC-MS) comprehensively profiles dynamic VOC signatures. Integrating these techniques enables comprehensive characterization of fungal spoilage at macroscopic, microscopic and molecular scales [11,12].

Orthogonal partial least squares discriminant analysis (OPLS-DA) paired with correlation analysis efficiently identifies infection-specific biomarkers to underpin fungal contamination early warning [13]. Against this backdrop, the present study established an *F. graminearum*-soybean meal contamination model via artificial inoculation. Over a 54-day incubation period, we continuously monitored fungal loads, microstructural morphology, protein secondary structures, and VOC profiles to unravel multi-layered deterioration patterns triggered by *F. graminearum* and screen strain-specific volatile biomarkers. Findings from this work enrich the existing theoretical basis of fungal-induced soybean meal spoilage and deliver scientific foundations and technical references for early detection, targeted mitigation and quality management of stored soybean meal.

## 2. Materials and Methods

### 2.1. Materials

Soybean meal was provided by Shenyang Fuhong Vegetable Oil Co., Ltd. (Shenyang, China) in June 2025 and stored at −4 °C before analysis.

### 2.2. Inoculation and Incubation of Soybean Meal with F. graminearum

Soybean meal inoculation assays were performed following the protocol of Gao et al. [14], with minor adjustments tailored to this study. Cryopreserved *F. graminearum* (glycerol stock solution preservation) was revived on PDA agar and incubated at 28 °C for 5 days to induce sporulation. The resultant fungal colonies were washed and transferred into CMC liquid medium, followed by shaking incubation at 25 °C and 180 rpm for 3–5 days to harvest conidia. A standardized conidial suspension at a concentration of 1 × 10^6^ conidia/mL was prepared for subsequent inoculation.

Prior to inoculation, 1000 g of soybean meal was sterilized and spread evenly into rectangular trays (40 cm × 25 cm). The conidial suspension was applied at a 1:10 solid–liquid weight–volume ratio (soybean meal:conidial suspension, *w*/*v*) and thoroughly blended with the substrate to achieve uniform fungal exposure. Trays were incubated in a programmable climate chamber (RHP-23, Huanrui, Dongguan, China) maintained at 30 °C and 70% relative humidity to mimic high-moisture warehouse storage conditions. The incubation trial spanned 54 days, with sampling conducted at 6-day intervals to generate ten time-point treatments: 0, 6, 12, 18, 24, 30, 36, 42, 48 and 54 days. At each sampling time, two 40 g subsamples were randomly collected. One subsample was immediately processed for fungal enumeration, while the other was vacuum freeze-dried (YT-DG30, Yutang, Weifang, China), pulverized and sieved through a 100-mesh screen. The processed powder was sealed and stored in the dark at −30 °C for subsequent SEM, FTIR, and GC-MS analyses. All experimental treatments were established in three independent biological replicates.

### 2.3. Determination of Total Mold Count in Soybean Meal

A total of 25 g of soybean meal sample was placed into a sterile homogenizing cup containing 225 mL of sterile phosphate buffer solution (pH 7.4, Iseju Biotechnology Co., Ltd., Lianyungang, China). After homogenization, a 1 mL sample homogenate was absorbed and injected into a test tube with 9 mL sterile phosphate buffer solution, followed by shaking and mixing on an oscillator to prepare 10-fold diluted sample homogenate. A 1 mL diluted sample suspension was absorbed with a sterile pipette and coated onto colony counting cards (Premium, Meizheng Biotechnology Co., Ltd., Rizhao, China). After incubation at 36 °C for 24 h, colony counting was performed using a colony counter (LB-6300, Loobo Co., Ltd., Qingdao, China).

### 2.4. SEM Analysis

Approximately 0.5 g of freeze-dried soybean meal powder at different infection stages was evenly adhered to conductive double-sided adhesive tape, subjected to ion sputtering gold coating treatment, and observed under a Hitachi S-4800 scanning electron microscope (Hitachi Ltd., Tokyo, Japan) with an accelerating voltage of 3.0 kV. The integrity, surface damage and hyphal growth adhesion of soybean meal protein particles and fiber skeletons under uninfected, slightly infected and severely infected conditions were compared and analyzed.

### 2.5. FTIR Analysis

The method reported by Wan et al. [15] was adopted with minor modifications. A total of 0.01 g of freeze-dried soybean meal powder was fully ground, mixed with 0.5 g of 200-mesh potassium bromide powder and pressed into uniform thin slices. Scanning was performed using a Nicolet iS5 FTIR spectrometer (Thermo Fisher Scientific, Waltham, MA, USA) within the band of 400–4000 cm^−1^ at a resolution of 4 cm^−1^ for 32 cumulative scans. PeakFit v4.12 software was used for deconvolution and second derivative peak fitting of the protein amide I band (1600–1700 cm^−1^) to quantitatively calculate the relative contents of four protein secondary structures: α-helix, β-sheet, β-turn and random coil.

### 2.6. GC-MS Analysis

GC-MS analysis was performed according to a modified method described by Ma et al. [16]. Headspace solid-phase microextraction (HS-SPME) combined with GC-MS was applied to determine volatile metabolites of soybean meal throughout the infection period. Accurately, 0.5 g of soybean meal sample was weighed into a 20 mL headspace vial, supplemented with 2.0 g NaCl and a quantitative internal standard 2,4,6-collidine (1000 ppm), then sealed and equilibrated in a water bath at 60 °C for 15 min. A 50/30 μm DVB/CAR/PDMS three-phase extraction fiber was selected and aged at 250 °C for 45 min, followed by headspace extraction at 60 °C for 40 min and thermal desorption at 250 °C in the injection port for 5 min with splitless injection.

Detection was performed on a TSQ 9000 triple quadrupole GC-MS instrument (Thermo Fisher Scientific, USA) equipped with an HP-5MS quartz capillary column (30 m × 0.25 mm × 0.25 μm). High-purity helium was used as carrier gas at a constant flow rate of 1.0 mL/min, and the injection port temperature was set at 250 °C. The temperature program was set as follows: hold at 40 °C for 3 min, ramp up to 150 °C at 5 °C/min, then ramp up to 250 °C at 10 °C/min and hold for 5 min, with a total running time of 40 min. Electron ionization (EI) mode was adopted for mass spectrometry with an ionization energy of 70 eV, an ion source temperature of 230 °C, a transfer line temperature of 280 °C, a solvent delay of 3 min, and a scanning range of m/z 35–550 under full-scan mode for data collection.

Qualitative identification of substances was conducted via retrieval from the NIST 21 standard spectral library, and double-verified with a forward matching degree ≥80% and a retention index (RI) of C5–C30 n-alkanes. Quantification was performed by the internal standard method, and the content of each volatile component (μg/kg) was calculated according to the peak area ratio of target substances to the internal standard.

### 2.7. Data Analysis

Three parallel determinations were carried out for all samples, and results were expressed as mean ± standard deviation (SD). All experiments were performed with three independent biological replicates to ensure data reproducibility. One-way analysis of variance (one-way ANOVA) was performed using SPSS 17.0 (SPSS Inc., Chicago, IL, USA), and *p* < 0.05 was defined as the criterion for significant inter-group difference. PeakFit v4.12 (SeaSolve Software Inc., San Jose, CA, USA) was used for peak fitting of the soybean meal amide I band and the calculation of relative contents of protein secondary structures. Origin 2024b (OriginLab, Northampton, MA, USA) was used for plotting, correlation analysis and cluster analysis. SIMCA 14.1 (Sartorius Aktiengesellschaft, Göttingen, Germany) software was applied for OPLS-DA. Orthogonal partial least squares discriminant analysis (OPLS-DA) was performed on unit-variance-scaled VOC datasets using SIMCA. The model contained 9 components, and model interpretability, goodness of fit and predictive ability were assessed via 7-fold cross-validation. A permutation test with 200 iterations was applied to diagnose overfitting. Differential VOCs were screened using a two-step strategy: (1) univariate comparison with FDR correction to control the false discovery rate; (2) variable importance in projection (VIP) derived from the OPLS-DA model. VOCs satisfying both FDR-adjusted significance and VIP > 1 were defined as differential volatile metabolites.

## 3. Results and Discussion

### 3.1. Dynamic Changes in Fungal Population and Protein FTIR Spectra During F. graminearum Infection

**Figure 1:**
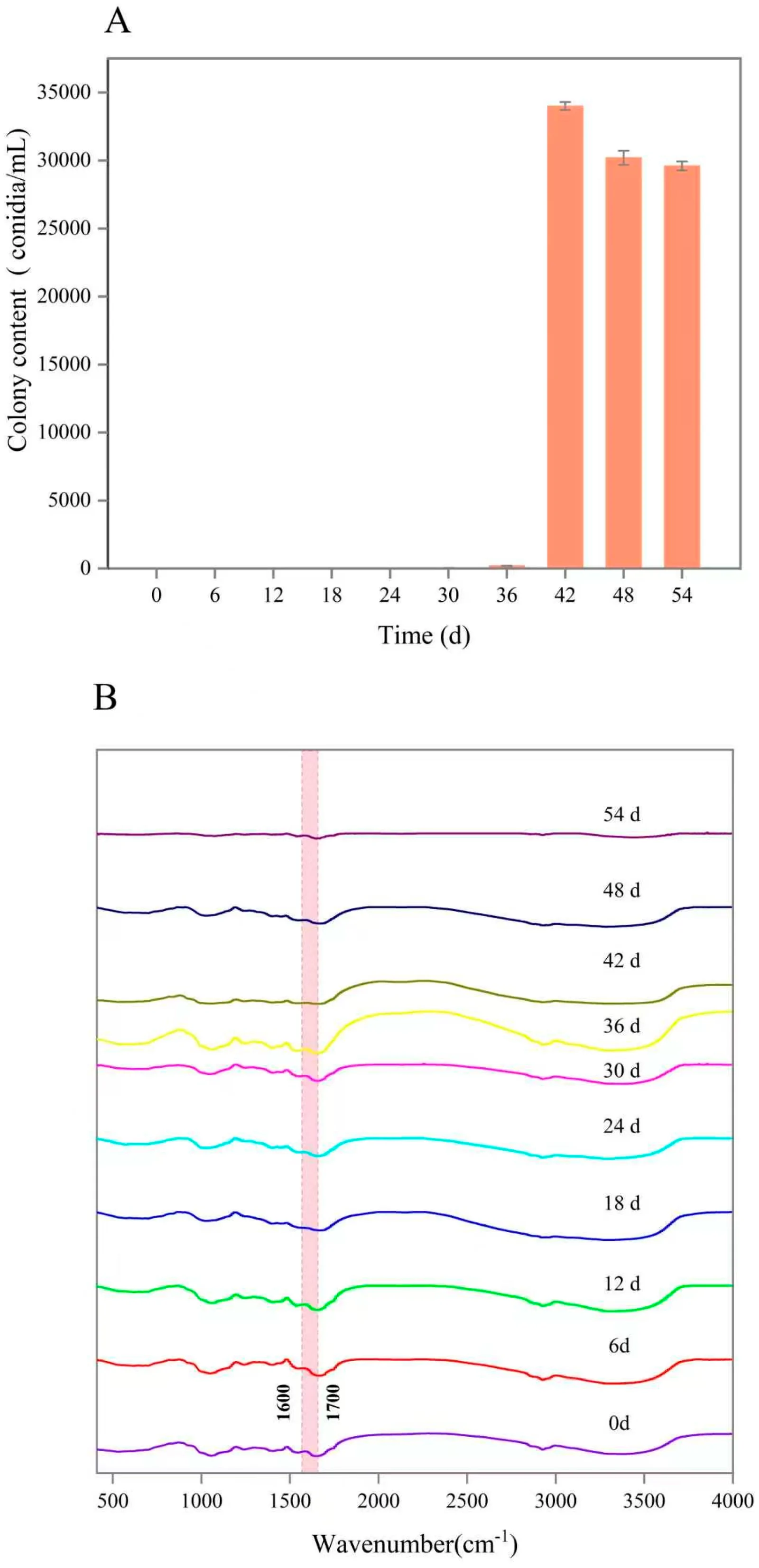
Dynamic variations in the fungal population and protein secondary structure of soybean meal during Fusarium graminearum infection over 54 days of storage. (**A**) Changes in *F. graminearum* conidia concentration (conidia/g) in soybean meal during the whole incubation period, showing a typical three-stage growth characteristic including latent infection, rapid proliferation, and decline-stabilization phases. (**B**) Fourier-transform infrared (FTIR) spectra of soybean meal protein, reflecting the dynamic alterations in protein molecular structure induced by continuous fungal infection.

The colony count of *F. graminearum* during the infection of soybean meal exhibited distinct stage-dependent dynamic variations, which could be divided into three phases: latent infection, rapid proliferation, and decline-stabilization stages (Figure 1A). The latent infection stage occurred from 0 to 30 d. During this period, the strain proliferated slowly in the soybean meal matrix, and the fungal colony count remained at an extremely low level (0 d: 0 conidia/g, 6 d: 2 ± 0.33 conidia/g, 12 d: 3 ± 0.33 conidia/g, 18 d: 5 ± 0.66 conidia/g, 24 d: 9 ± 0.66 conidia/g, 30 d: 37 ± 0.66 conidia/g). This phenomenon was primarily attributed to the unbalanced water status between the soybean meal substrate and the external storage environment. The strain mainly completed spore germination, hyphal colonization, and environmental adaptation, with no obvious deterioration in the apparent quality of soybean meal. We defined 36 d as the critical inflection point marking the onset of accelerated fungal proliferation, where the growth rate sharply increased and massive hyphal expansion initiated, accompanied by the start of irreversible quality deterioration of soybean meal. Notably, the fungal population continued to accumulate after 36 d and finally reached the maximum colony concentration at 42 d, representing the peak of the entire infection cycle. Subsequently, the fungal abundance slightly decreased and gradually stabilized due to substrate nutrient depletion, accumulation of metabolic by-products, and microbial living stress, which is consistent with the typical growth succession rule of fungi on grain and oil substrates [17].

Characteristic peaks of the protein amide I band (1600–1700 cm^−1^) in Figure 1B could directly reflect molecular conformational damage of soybean meal protein under the stress of *F. graminearum* infection. During uninfected and early infection stages (0–30 d), hydrogen bond structures of protein molecules remained stable with no significant change in absorption intensity of the amide I band. After day 36, massive proliferation of *F. graminearum* and secretion of extracellular hydrolases continuously broke peptide bonds and destroyed the spatial structure of protein molecules, leading to a significant decline in the absorption intensity of the amide I band, as well as irreversible oxidative degradation and conformational damage of ordered protein structures [18].

Deconvolution and second derivative processing of the amide I band yielded the relative contents of α-helix, β-sheet, β-turn and random coil of soybean meal protein, as shown in Table 1.

Quantitative results of protein secondary structures indicated that exogenous infection of *F. graminearum* significantly induced disordered conformational transformation of soybean meal protein. With prolonged infection time, ordered structures maintaining stable protein spatial conformation continuously declined: the relative content of α-helix decreased from an initial 53.60% to 45.61% at day 54, while β-sheet dropped from 13.24% to 10.08%. In contrast, the proportions of disordered structures β-turn and random coil increased significantly from 13.51% and 19.65% to 19.32% and 24.99%, respectively. The underlying mechanism was that oxidases and hydrolases secreted during *F. graminearum* proliferation continuously destroyed hydrogen bonds and hydrophobic interactions of protein molecules, resulting in the unfolding of ordered spatial structures, increased flexibility and loss of stability, which constituted the core molecular mechanism of functional and nutritional quality deterioration of soybean meal after infection [19,20].

### 3.2. Microstructural Variations in Soybean Meal Infected by F. graminearum

**Figure 2:**
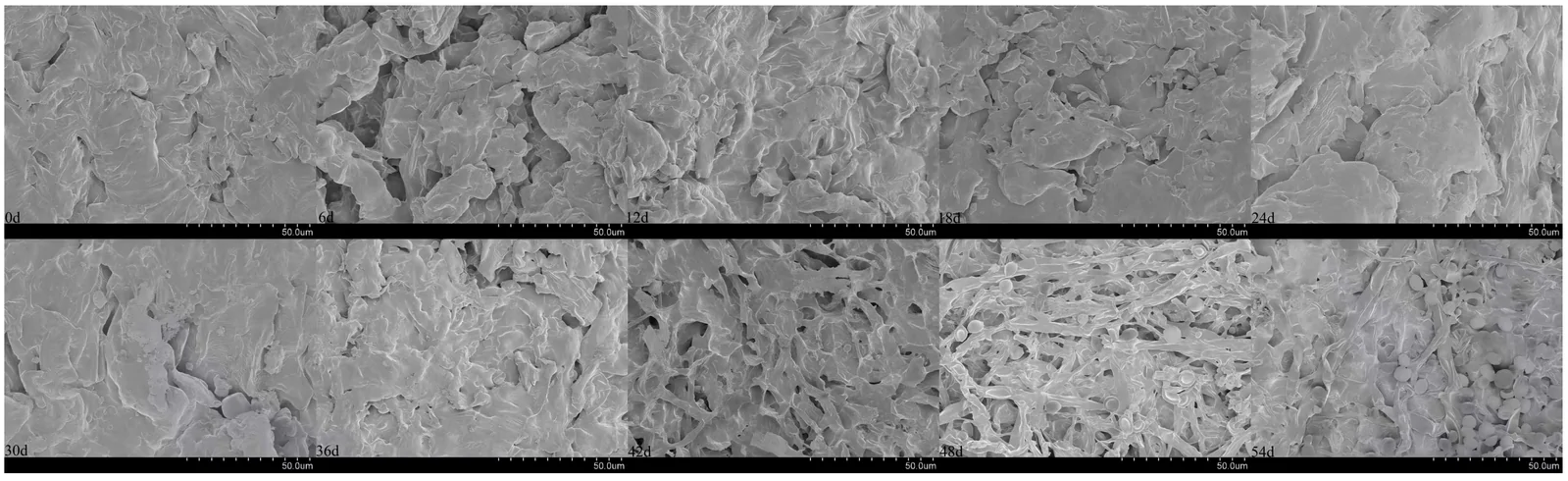
Microstructure of soybean meal during *F. graminearum* infection.

Scanning electron microscopy (SEM) revealed pronounced micromorphological alterations in soybean meal substrates during sequential Fusarium graminearum infection under simulated storage conditions (Figure 2). In the uninfected control and the 0–30 d latent infection stage, soybean meal maintained intact and well-organized intrinsic tissue morphology. Protein granules exhibited full, rounded shapes with clear and intact boundaries, which were tightly cross-linked and embedded within fibrous cellulose networks. The overall substrate presented a dense, homogeneous, and compact microstructure without obvious crevices, cavities, or fragmentation, indicating well-preserved physical integrity and negligible fungal colonization during the early incubation period.

In the rapid deterioration stage at day 36, extensive hyphal proliferation of *F. graminearum* was clearly observed on soybean meal surfaces. Abundant hyphae adhered tightly to the outer substrate layers and further penetrated into the internal matrix. Hydrolases continuously secreted by colonizing mycelia triggered progressive degradation of structural proteins and cell wall polysaccharides. Macromolecular breakdown generated numerous microcavities and fissures throughout the substrate, gradually loosening the original compact structure and disrupting the homogeneous microstructure of soybean meal.

During the severe spoilage stage (42–54 d), dense hyphal networks fully covered soybean meal particles. The originally rigid and compact substrate framework underwent severe collapse and disaggregation. Large quantities of protein matrices fragmented into irregular, coarse, and loosely aggregated debris, and the integrated tissue architecture was thoroughly destroyed, leading to irreversible deterioration of substrate structural integrity [21].

The progressive microstructural degradation observed via SEM exhibited high temporal consistency with the dynamic trends of fungal population proliferation and protein secondary structural disorder. These synchronous morphological and biochemical changes collectively demonstrate that continuous *F. graminearum* colonization and metabolic secretion serve as the primary drivers of microstructural collapse and comprehensive quality deterioration in soybean meal under high-humidity storage conditions.

### 3.3. Composition Characteristics of VOC in Soybean Meal

**Figure 3:**
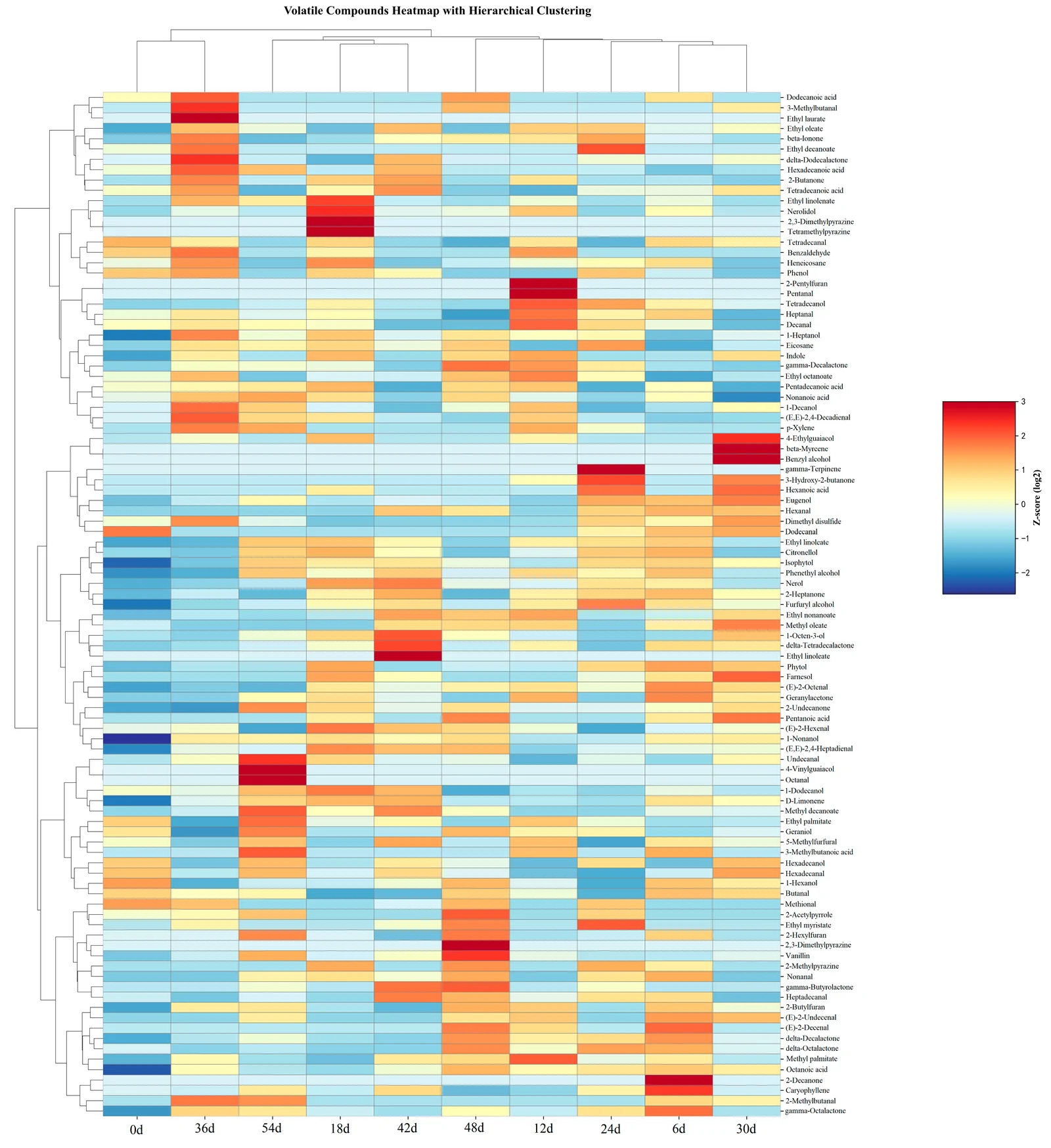
Heatmap of normalized volatile organic compound (VOC) abundances in soybean meal during *F. graminearum* spoilage. Rows represent individual VOCs; columns represent sampling time points after inoculation. Color gradient corresponds to Z-score-normalized VOC abundance (red = higher abundance, blue = lower abundance). Hierarchical clustering was performed using Euclidean distance.

Headspace solid-phase microextraction coupled with gas chromatography–mass spectrometry (HS-SPME-GC-MS) was adopted to comprehensively profile volatile organic compounds (VOCs) released from soybean meal throughout the 54-day incubation period under artificial *F. graminearum* contamination (Figure 3, Table S1). In total, 99 individual VOCs were successfully identified and classified into 13 distinct chemical families, namely, 22 aldehydes, 15 alcohols, 13 esters, 7 ketones, 9 carboxylic acids and furans, 5 phenols, 4 pyrazines, 3 aliphatic and aromatic hydrocarbons, 6 terpenes, 7 lactones, 2 nitrogen heterocyclics, and 2 sulfur-containing derivatives. These chemical groups cover two core origins of volatile metabolites in spoiled agricultural commodities: primary oxidation products derived from endogenous lipid and protein degradation of soybean meal matrix, and secondary characteristic off-odor metabolites biosynthesized by the growing fungal mycelia, which is consistent with previous research on volatile signatures of mold-contaminated grain substrates [22].

Soybean meal without fungal inoculation exhibited a relatively simple and stable volatile spectrum dominated by mild, native aromatic compounds originating from raw soybean lipids and amino acids. Undesirable malodorous compounds associated with spoilage were barely detectable in the control group, indicating intact nutritional and flavor properties of fresh soybean meal before storage fungal infestation. With the prolonged colonization and metabolic activity of *F. graminearum*, massive microbe-specific volatile metabolites were continuously synthesized and accumulated within the substrate system. Among all detected VOC categories, aldehydes, alcohols and furans exhibited far more drastic concentration fluctuations across all infection time points and showed the most remarkable sensitivity to fungal proliferation. For this reason, these three chemical classes can be regarded as core volatile biomarkers to discriminate the health status of stored soybean meal and quantitatively reflect the progressive degree of quality deterioration triggered by *F. graminearum* [23].

The hierarchical clustering heatmap generated based on normalized VOC abundances further visualized the holistic divergence of volatile fingerprints across different infection stages. Samples collected within the latent infection window (0–30 d) clustered closely together and shared highly analogous volatile composition, which corresponded to the slow and limited metabolic activity of dormant fungal spores and immature hyphae at this stage. In contrast, substantial remodeling of the overall volatile profiles took place once the substrate entered the rapid deterioration phase (36 d) and subsequent severe spoilage stage (42–54 d). Large shifts in the relative proportions of innate soybean flavor compounds and newly produced fungal off-flavors clearly separated severely contaminated samples from mildly infected and healthy controls, demonstrating that VOC fingerprinting can effectively track the continuous spoilage progression of stored soybean meal.

### 3.4. Stage Classification and Differential VOC Screening via Principal Components Analysis (PCA) and OPLS-DA Model

**Figure 4:**
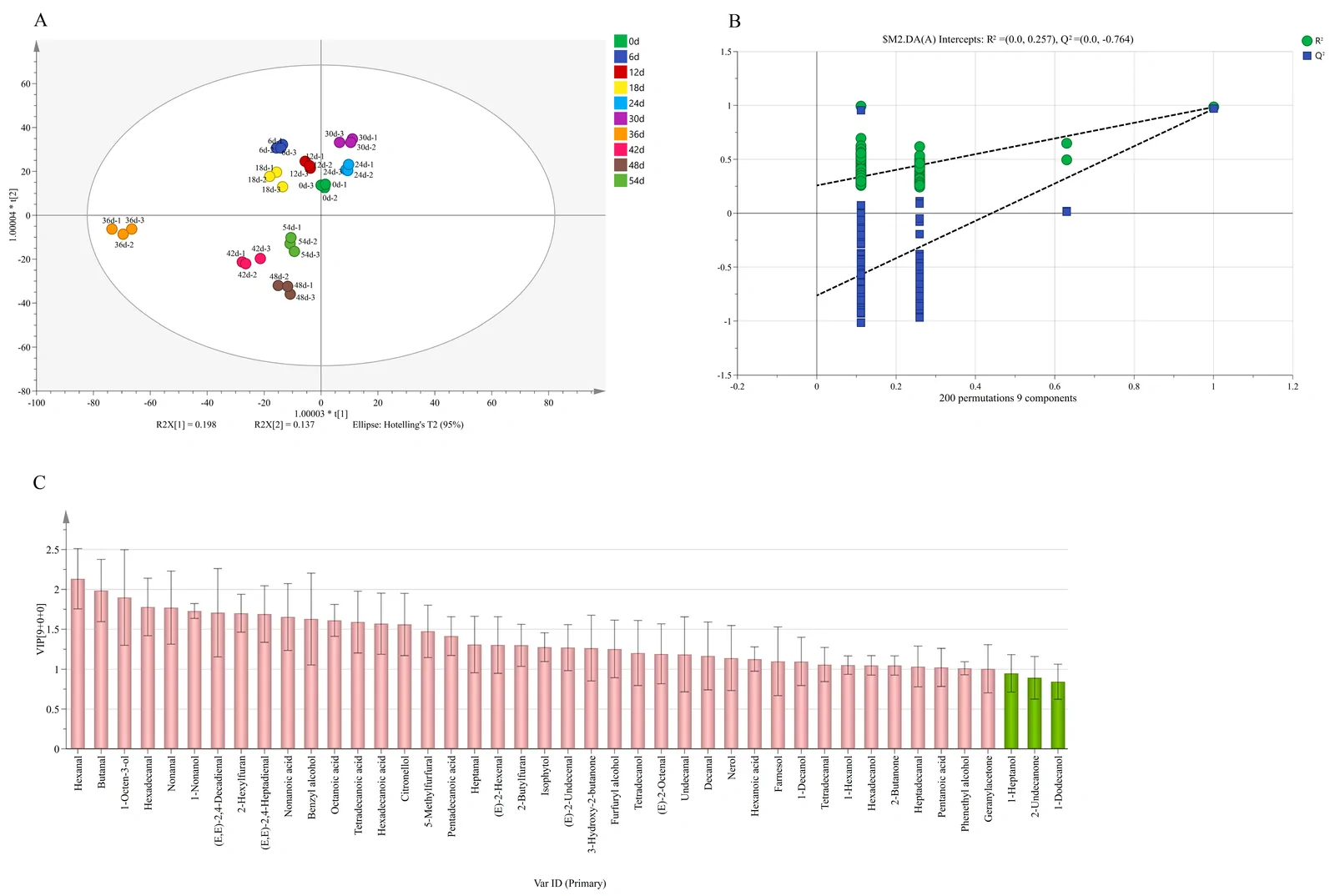
PCA overview and OPLS-DA-based variable screening of volatile organic compounds (VOCs) in soybean meal during Fusarium graminearum infection. (**A**) PCA score plot with Hotelling’s T ellipse (95% confidence interval), showing sample separation across different infection time points; R^2^X[1] = 0.198, R^2^X[2] = 0.137. (**B**) Permutation validation plot based on 200 permutations for the 9-component OPLS-DA model, with intercepts R^2^ = 0.257 and Q^2^ = −0.764. (**C**) VIP plot of all VOCs; variables with VIP > 1 were screened as differential VOCs after FDR-adjusted univariate significance testing.

The resulting PCA score plot (Figure 4A) exhibited obvious grouping separation across the entire 54 d incubation trial, successfully partitioning all samples into three distinct clusters that perfectly matched the three-stage deterioration pattern observed in previous phenotypic indicators. Specially, all specimens collected from 0 to 30 d were tightly aggregated into the first cluster, which corresponded to the latent infection phase with negligible fungal growth and intact soybean meal quality. Samples harvested at 36 d formed an isolated intermediate group, marking the critical transition inflection point where fungal metabolism abruptly intensified and irreversible substrate deterioration initiated. Specimens from 42 to 54 d were fully separated into the third cluster, representing the advanced severe spoilage phase with massive hyphal accumulation and thorough destruction of soybean meal matrix. Such clear sample grouping derived from VOC profiles was highly congruent with the staged dynamic trends of fungal colony abundance, protein secondary structural disorder and microstructural collapse described in the preceding sections, further verifying that volatile metabolite fingerprints can objectively reflect the progressive spoilage severity of stored soybean meal.

To quantitatively distinguish soybean meal samples with varying degrees of *F. graminearum* contamination and screen differential volatile metabolites, orthogonal partial least squares discriminant analysis (OPLS-DA) was performed on the full set of normalized VOC quantitative data via SIMCA 14.1 software. Permutation testing with 200 random iterations was further implemented to cross-validate the reliability and predictive performance of the established OPLS-DA classification model (Figure 4B). The cross-validation parameter Q^2^ was calculated as −0.764, indicating no severe overfitting; model stability and predictive performance were further evaluated via 7-fold cross-validation. On the basis of the validated model, variable importance in projection (VIP) values were extracted to screen candidate volatile biomarkers, with VIP > 1 set as the screening threshold combined with one-way ANOVA significance (*p* < 0.05). A total of 39 differential VOCs satisfying both statistical criteria were ultimately identified (Figure 4C). The relative concentrations of these differential metabolites fluctuated drastically across latent, rapid deterioration and severe spoilage periods, revealing prominent stage-specific variation characteristics and demonstrating great application potential as characteristic indicators to identify fungal contamination in soybean meal. Collectively, the OPLS-DA modeling strategy not only achieved precise automatic classification of soybean meal samples according to contamination severity, but also sharply reduced the range of candidate volatile compounds from the original 99 total VOCs to 39 differential variables. This greatly simplified the subsequent screening workflow for strain-specific characteristic biomarkers and laid a solid multivariate data foundation for the final identification of highly specific volatile markers for the early warning of *F. graminearum* contamination in stored soybean meal [24].

### 3.5. Screening of Specific Volatile Markers for F. graminearum Infection

**Figure 5:**
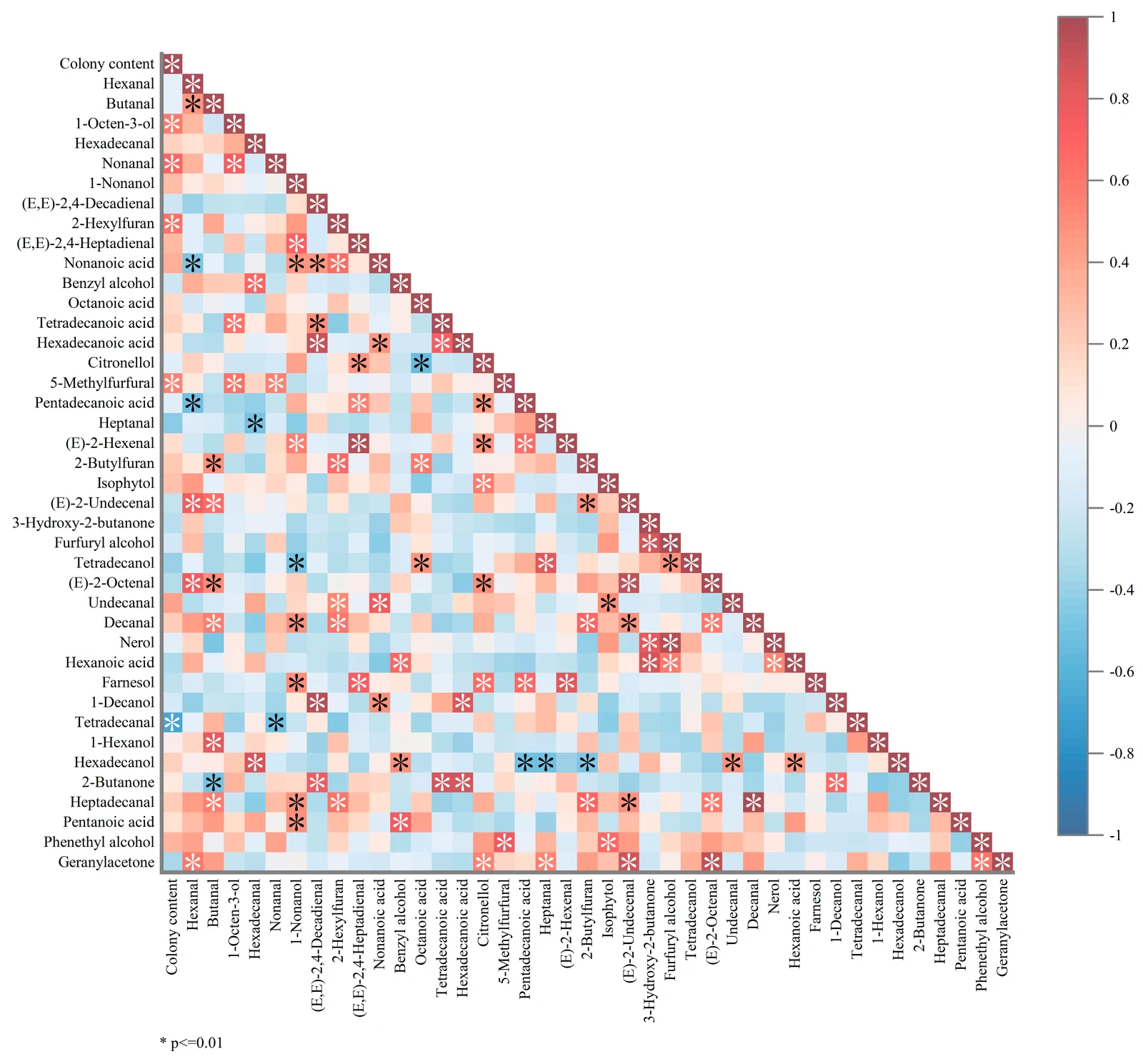
Analysis of the correlation between differential VOC and total mold count after *F. graminearum* infection. The five candidate VOCs were screened by Pearson correlation analysis; their specificity and predictive performance require further independent external validation.

To further screen representative volatile biomarkers closely associated with *F. graminearum* contamination severity, Pearson correlation analysis was conducted between the quantitative abundances of the 39 OPLS-DA-screened differential VOCs and dynamic fungal population counts across all incubation time points (Figure 5). Compounds with extremely significant correlations (*p* < 0.01) were retained as candidate biomarkers. Through this two-step screening strategy combining multivariate classification and univariate correlation analysis, five volatile compounds with potential diagnostic characteristics for *F. graminearum* infestation were finally identified: 1-octen-3-ol, nonanal, 2-hexylfuran, 5-methylfurfural, and tetradecanal.

Four of these five target compounds—1-octen-3-ol, nonanal, 2-hexylfuran and 5-methylfurfural—shared an identical variation trend and showed extremely significant positive correlations with fungal population size, with their concentrations rising persistently as the fungal incubation period extended and spoilage aggravated. As a classic fungal-specific secondary metabolite, 1-octen-3-ol emits distinctive musty and mushroom-like off-odors that are universally recognized as markers of mold spoilage in stored oilseed substrates. This compound is biosynthesized when fungal lipoxygenases catalyze the oxidative cleavage of linoleic acid released from soybean cell membrane lipids [25]. Nonanal is responsible for unpleasant rancid and earthy odors in deteriorated feed materials, originating from the autoxidation and microbial degradation of endogenous unsaturated fatty acids in soybean meal [26]. Meanwhile, 2-hexylfuran and 5-methylfurfural deliver noticeable burnt aromas, which are signature by-products of lipid peroxidation triggered by fungal oxidative enzymes. Their accumulated levels can serve as intuitive quantitative indicators to evaluate the degree of lipid oxidative deterioration occurring in stored soybean meal under high-humidity conditions [27].

In contrast, tetradecanal displayed an opposite variation pattern and had an extremely significant negative correlation with total fungal counts, with its concentration declining steadily throughout the entire 54 d infection trial. Tetradecanal is an inherent native lipid-derived flavor compound originating from fresh soybean meal, contributing to the mild natural aroma of uninfected feed raw materials. During sustained fungal colonization, diverse hydrolases and oxidases secreted by proliferating *F. graminearum* continuously break down large lipid macromolecules in the substrate, resulting in the constant decomposition and consumption of tetradecanal, which explains its progressive concentration loss as mold contamination worsens.

The coordinated but opposite variation patterns of these five VOCs effectively characterize the dynamic spoilage progression of *F. graminearum*-contaminated soybean meal under laboratory-controlled conditions. The unique volatile fingerprint consisting of four infection-induced upregulated metabolites and one downregulated endogenous flavor compound provides preliminary laboratory data and a theoretical reference for exploring VOC-based spoilage monitoring methods. It should be noted that the reliability and universality of this biomarker fingerprint are only verified under simplified laboratory single-strain inoculation conditions; further external validation under complex industrial storage environments is still required for practical application.

## 4. Conclusions

The quality deterioration of Fusarium graminearum-infected soybean meal over the 54-day simulated storage cycle exhibited distinct three-stage dynamic characteristics: a latent infection stage (0–30 d), a rapid deterioration inflection stage (36 d), and a severe spoilage stage (42–54 d). The latent stage was characterized by suppressed fungal proliferation, with no remarkable damage to the protein secondary structure or the microscopic morphology of soybean meal. After day 36, explosive fungal growth triggered continuous degradation of ordered protein conformations and progressive collapse of the substrate microstructural skeleton. Fungal conidia abundance peaked at day 42 and declined slightly thereafter, accompanied by the irreversible and severe structural and compositional deterioration of soybean meal.

*F. graminearum* infection significantly reshaped the protein secondary structure of soybean meal during incubation. As contamination progressed, the relative proportions of ordered α-helix and β-sheet structures decreased continuously, whereas the contents of disordered β-turn and random coil structures increased markedly. The disruption of intramolecular hydrogen bonds by fungal extracellular enzymes is the primary driver of protein conformational disorder and the structural destabilization of soybean meal substrates.

A total of 99 volatile organic compounds (VOCs) belonging to 13 chemical classes were identified throughout the infection process, among which, aldehydes, alcohols, and furans exhibited the most sensitive responses to fungal colonization. The validated OPLS-DA model achieved accurate classification of samples corresponding to the three deterioration stages, with no obvious overfitting, and successfully screened 39 differential VOCs with stage-specific variation patterns. Further combination with Pearson correlation analysis enabled the final identification of five core volatile biomarkers tightly associated with *F. graminearum* contamination severity. Four metabolites (1-octen-3-ol, nonanal, 2-hexylfuran, and 5-methylfurfural) were continuously upregulated with aggravated spoilage, while the endogenous flavor compound tetradecanal was progressively downregulated, forming a unique discriminatory volatile fingerprint under laboratory-controlled conditions.

Combined with Pearson correlation analysis, five specific volatile markers closely associated with *F. graminearum* contamination were successfully screened. Among them, four metabolites (1-octen-3-ol, nonanal, 2-hexylfuran, and 5-methylfurfural) were continuously upregulated with the aggravation of fungal spoilage, while the endogenous substance tetradecanal was persistently downregulated. Based on these differential VOC profiles, a characteristic flavor fingerprint was established for the early identification of *F. graminearum* contamination in soybean meal. This study provides a feasible technical basis for rapid and non-destructive monitoring of soybean meal quality. Future studies will focus on verifying the universality and stability of these VOC markers under actual storage conditions, optimizing the fingerprint model, and further developing portable detection strategies, aiming to promote this approach as a reliable non-destructive analytical tool for industrial soybean meal safety supervision.

This study systematically elucidated the multi-dimensional deterioration mechanism of soybean meal under *F. graminearum* stress, integrating fungal population dynamics, protein conformational changes, microstructural disruption, and volatile metabolic profiling. The screened VOC biomarkers provide preliminary laboratory evidence and a theoretical reference for the potential development of non-destructive detection and spoilage monitoring strategies for soybean meal fungal contamination.

Notably, this study has clear limitations, and we further justify the rationality and applicability of the adopted laboratory model as follows. The sterile and single-species *F. graminearum* artificial inoculation system under fixed laboratory conditions was deliberately employed in this study. This simplified experimental model eliminates the interference of indigenous microorganisms, variable substrate backgrounds, and fluctuating environmental factors in industrial environments, making it possible to independently clarify the single-effect deterioration mechanism and exclusive volatile metabolic characteristics triggered by *F. graminearum* colonization in soybean meal. It provides purified, controllable, and repeatable baseline data for exploring fungal-induced spoilage characteristics, which is a necessary prerequisite for subsequent complex multi-factor verification.

Nevertheless, the findings of this controlled exploratory study cannot be directly extrapolated to practical industrial storage scenarios. Actual industrial soybean meal storage constitutes a complex open ecosystem, which contains diverse indigenous microflora, inevitable interspecific microbial competition and synergistic effects, inconsistent raw material quality, and dynamic fluctuations in temperature and humidity. These complex field factors may reshape microbial metabolic patterns, alter volatile metabolite profiles, and interfere with the specificity and stability of the screened VOC biomarkers.

Accordingly, the current work serves only as a fundamental laboratory exploration focusing on the single-pathogen-induced spoilage mechanism. Future studies will abandon the sterile single-strain system, adopt non-sterile natural substrates and mixed microbial inoculation, and conduct simulated warehouse storage trials with variable environmental parameters. These external validations will further verify the universality and robustness of the VOC fingerprint model, optimize the biomarker screening system, and ultimately improve the feasibility of this monitoring strategy for industrial soybean meal safety supervision.

## Figures and Tables

**Table 1:** The relative content of secondary structure of protein in soybean meal after infection.

	α-Helix/%	β-Pleated Sheet/%	β-Turn/%	Random Coil/%
0 d	53.60 ^a^*	13.24 ^b^	13.51 ^e^	19.65 ^de^
6 d	53.84 ^a^	13.61 ^a^	14.45 ^d^	18.12 ^f^
12 d	52.10 ^b^	14.60 ^a^	14.69 ^d^	18.61 ^f^
18 d	52.08 ^b^	14.03 ^a^	14.81 ^d^	19.09 ^e^
24 d	51.37 ^c^	13.97 ^a^	15.63 ^c^	19.03 ^e^
30 d	50.59 ^cd^	12.93 ^b^	15.96 ^c^	20.52 ^d^
36 d	49.59 ^d^	12.23 ^c^	16.58 ^b^	21.60 ^c^
42 d	47.20 ^e^	11.81 ^d^	17.98 ^b^	23.01 ^b^
48 d	46.06 ^f^	10.21 ^e^	19.25 ^a^	24.48 ^a^
54 d	45.61 ^g^	10.08 ^e^	19.32 ^a^	24.99 ^a^

* Statistical differences were analyzed using one-way ANOVA with Duncan’s multiple range post hoc test (*p* < 0.05). Superscript letters denote statistical significance: values sharing identical letters within a column are not significantly different, whereas values marked with different letters differ significantly.

## Data Availability

The original contributions presented in this study are included in the article/Supplementary Material. Further inquiries can be directed to the corresponding authors.

## Supplementary Materials

The following supporting information can be downloaded at: https://www.mdpi.com/article/10.3390/foods15173009/s1, Supplementary Table S1. Analysis of VOCs released from soybean meal artificially inoculated with *F. graminearum* during the 54 d incubation period.
