# Supplementary material for: Dynamic Deterioration Pattern of Soybean Meal Contaminated by Fusarium graminearum
Source: Foods. 2026 Aug 26;15(17):3009. doi: 10.3390/foods15173009 (PMC13565134; doi:10.3390/foods15173009)
Supplement: Supplementary file 1 [file foods-15-03009-s001.zip › foods-4497321-supplementary.pdf]

Supplementary Table Caption

Supplementary Table S1. Analysis of VOCs released from soybean meal artificially inoculated with *F. graminearum* during the 54 d incubation period.

Compound name	Types	Content of different storage times (μg/kg)									
		0d	6d	12d	18d	24d	30d	36d	42d	48d	54d
Butanal		390.02±40.70	568.49±122.65	71.40±2.87	4.49±0.46	5.87±0.25	344.44±32.66	100.35±8.28	6.81±0.71	416.89±88.43	146.45±12.63
3-Methylbutanal		–	–	–	–	–	5.01±0.47	119.19±7.81	–	16.99±1.05	–
2-Methylbutanal		–	15.87±1.65	–	–	–	6.82±0.35	90.97±13.60	–	–	59.46±4.50
Pentanal		–	–	13.79±1.72	–	–	–	–	–	–	–
Hexanal		–	727.00±38.92	–	–	270.84±13.31	462.79±71.12	–	345.83±83.77	122.17±6.56	–
(E)-2-Hexenal		39.02±3.96	32.93±1.45	45.52±5.57	300.46±24.12	9.28±0.97	48.49±10.76	49.56±2.95	152.93±5.43	117.65±6.86	9.05±1.35
Heptanal		38.66±2.24	109.88±5.25	270.46±20.01	52.73±3.90	61.35±6.14	9.42±0.45	78.57±3.19	18.62±0.92	6.87±1.22	30.05±1.14
Benzaldehyde		18.81±3.27	–	46.93±4.55	6.66±0.54	–	–	83.71±13.98	–	–	–
Octanal		–	–	–	–	–	–	–	–	–	7.06±0.93
(E,E)-2,4-Heptadienal		8.85±1.61	65.50±6.63	24.22±3.75	471.70±20.34	53.92±0.17	75.65±13.85	63.15±4.91	269.97±20.07	288.29±43.55	50.64±8.49
(E)-2-Octenal	Aldehydes	4.72±0.58	264.05±43.31	94.31±2.37	83.63±3.90	37.50±2.76	106.05±18.78	9.21±1.19	31.99±2.90	63.98±7.60	6.42±0.43
Heptadecanal		–	141.93±12.29	–	43.49±6.37	41.97±2.32	–	–	8.92±0.81	146.34±11.45	22.41±2.25
Decanal		11.03±0.23	7.26±0.70	277.92±30.81	10.23±0.52	39.71±2.57	–	30.61±4.85	–	–	14.38±1.16
(E)-2-Decenal		–	20.22±0.31	4.58±0.55	–	–	–	–	–	15.72±1.37	–
U–ecanal		12.00±1.57	9.43±0.39	6.02±0.74	55.72±3.26	19.16±2.24	32.63±4.02	26.68±3.31	17.57±0.54	19.81±2.99	223.69±20.61
(E,E)-2,4-Decadienal		12.83±2.13	3.90±0.20	110.94±7.54	68.58±6.78	8.41±0.70	5.36±0.78	561.36±66.83	9.12±1.15	4.56±0.41	89.32±11.85
(E)-2-U–ecenal		–	258.63±37.72	95.72±7.15	–	–	131.60±16.69	–	–	48.53±3.40	30.50±3.19
Dodecanal		46.53±6.35	17.36±1.36	–	–	5.23±0.83	24.48±4.41	–	–	–	–
Tetradecanal		135.48±9.78	86.84±5.53	71.70±4.24	87.86±12.03	7.34±1.51	57.03±9.70	60.83±6.68	11.77±0.37	6.76±0.39	11.87±0.62
Pentadecanal		39.89±4.16	35.16±4.60	51.96±4.38	197.91±26.70	28.84±2.30	243.44±13.62	28.95±1.04	629.22±12.05	98.72±21.13	80.61±8.07
Hexadecanal		260.80±38.39	25.12±1.60	8.06±0.37	30.42±4.75	5.19±0.55	443.34±21.42	21.16±2.41	185.70±25.68	33.88±3.88	310.53±47.74
Nonanal		35.75±4.18	161.65±12.96	50.40±5.07	21.14±0.67	148.28±10.37	14.70±2.29	15.64±2.25	497.99±28.49	281.00±37.88	40.94±2.46

Ethyl acetate		–	–	–	–	–	–	–	45.46±3.59	–	–
Ethyl octanoate		6.98±0.75	0.88±0.09	41.36±4.20	5.16±0.66	9.59±0.32	3.42±0.22	25.05±1.81	4.49±0.70	23.20±3.56	1.77±0.10
Ethyl nonanoate		1.23±0.13	3.08±0.41	16.41±0.89	2.58±0.25	2.39±0.36	10.38±0.75	2.74±0.29	16.78±1.07	12.14±0.34	2.04±0.03
Methyl decanoate		–	1.41±0.06	–	2.42±0.32	–	1.02±0.03	0.66±0.12	14.66±1.59	2.21±0.23	21.93±2.56
Ethyl decanoate		0.74±0.02	–	–	–	19.85±3.73	–	14.00±0.84	–	–	–
Ethyl laurate	Ester	–	–	–	–	–	–	13.67±1.94	–	–	–
Ethyl myristate		–	–	–	–	47.66±5.37	–	3.69±0.38	1.87±0.09	27.50±0.63	–
Methyl palmitate		2.72±0.33	25.25±3.70	110.47±14.39	3.20±0.19	11.96±1.20	6.89±0.74	18.98±2.55	26.02±2.54	33.23±4.11	5.66±0.22
Ethyl palmitate		27.71±4.66	2.75±0.25	31.50±0.78	7.02±0.75	8.08±0.99	3.91±0.54	0.67±0.07	13.11±0.39	2.13±0.06	82.09±7.48
Methyl oleate		0.76±0.05	3.59±0.12	5.93±0.69	–	–	14.18±2.08	–	5.31±0.49	5.49±0.97	–
Ethyl oleate		1.15±0.09	7.27±0.58	31.50±3.61	1.80±0.19	32.62±0.17	11.99±2.48	39.87±3.68	40.37±4.63	1.93±0.07	9.31±0.62
Ethyl linoleate		0.74±0.03	44.65±6.74	22.89±4.54	51.10±9.62	33.40±6.14	4.22±0.57	1.35±0.19	19.16±1.34	2.52±0.24	48.58±4.53
Ethyl linolenate		–	1.90±0.19	2.23±0.17	90.75±4.50	–	–	20.08±1.93	0.54±0.04	–	6.29±0.27
2-Butanone		2.42±0.06	3.07±0.36	34.71±3.39	56.78±2.33	2.34±0.23	1.08±0.04	182.19±9.64	122.89±6.99	2.02±0.37	3.85±0.04
3-Hydroxy-2-butanone		–	–	3.72±0.46	–	213.70±32.62	75.89±8.78	–	–	–	–
2-Heptanone		–	38.73±3.63	13.27±1.48	11.71±1.28	25.43±3.61	9.35±0.77	2.29±0.15	49.04±5.37	–	–
2-Decanone	Ketones	–	2.99±0.25	–	–	–	–	–	–	–	–
2-U–ecanone		1.46±0.24	18.37±2.22	10.79±2.09	48.16±2.66	10.59±1.17	45.30±5.90	1.00±0.08	12.02±0.98	41.41±3.72	122.63±2.94
Geranylacetone		–	164.10±17.67	77.79±2.96	31.44±3.29	–	21.41±0.88	–	3.58±0.20	–	11.37±0.87
beta-Ionone		1.50±0.03	5.43±0.53	21.17±2.91	2.36±0.18	52.77±5.87	2.51±0.15	79.66±10.31	10.93±1.52	16.10±2.68	1.03±0.09
p-Xylene		–	–	7.63±0.34	–	1.29±0.06	–	12.30±2.09	–	–	8.13±0.29
Eicosane	Hydrocarbons	2.15±0.19	1.14±0.03	1.55±0.06	16.29±2.17	28.29±1.91	3.91±0.16	14.43±2.36	6.36±0.78	17.83±0.87	10.16±0.54
Heneicosane		2.19±0.20	7.72±0.68	2.53±0.19	19.98±4.04	3.84±0.59	–	18.90±1.71	–	0.97±0.17	–
3-Methylbutanoi	Acids	–	18.65±2.88	13.85±1.81	–	–	–	–	–	–	55.37±10.01

c acid											
Pentanoic acid		–	11.68±1.97	–	11.02±0.32	–	117.84±8.62	–	–	93.45±11.95	–
Hexanoic acid		–	–	–	8.04±0.20	126.51±8.03	132.46±12.01	–	–	–	–
Octanoic acid		1.93±0.05	232.20±30.28	85.96±9.94	17.30±2.22	141.62±10.48	112.35±4.27	79.56±2.27	57.01±2.61	303.46±20.78	22.85±1.78
Nonanoic acid		47.25±4.33	86.55±13.84	45.74±7.45	186.93±10.09	15.61±1.42	4.23±0.32	290.10±26.05	14.33±0.81	215.20±19.73	439.12±43.81
Dodecanoic acid		4.24±0.35	10.55±1.63	–	–	–	–	110.60±5.32	–	40.49±3.56	–
Tetradecanoic acid		58.89±2.69	45.05±3.90	7.91±1.06	85.10±4.69	44.39±4.26	124.84±7.98	344.10±66.44	385.08±43.67	12.21±1.48	8.74±1.00
Pentadecanoic acid		23.85±3.56	30.65±0.82	215.95±15.66	300.77±23.35	–	–	44.59±3.99	–	129.26±8.64	77.35±9.23
Hexadecanoic acid		26.94±2.97	5.22±0.44	13.03±1.79	12.74±0.36	13.52±1.83	9.49±0.49	458.06±15.47	157.97±25.03	12.51±2.36	134.69±15.32
Furfuryl alcohol		–	59.17±7.72	43.40±1.93	30.21±0.41	234.35±13.01	18.12±1.46	4.05±0.48	60.25±1.26	7.82±0.64	8.21±1.22
1-Hexanol		124.21±7.90	77.69±11.26	13.05±1.09	9.46±0.41	2.21±0.24	38.46±7.58	2.54±0.47	18.79±3.65	91.28±7.28	10.13±0.87
1-Heptanol		1.16±0.20	4.38±0.90	27.16±0.70	85.04±8.61	33.30±5.25	15.49±1.99	172.22±22.34	14.87±0.64	52.38±10.31	21.46±1.23
Benzyl alcohol		–	–	–	–	–	336.56±35.71	–	–	–	–
Phenethyl alcohol		0.88±0.10	122.08±10.47	82.91±5.73	25.22±1.01	38.98±1.80	8.10±0.84	1.96±0.22	99.06±14.71	11.99±1.73	103.36±15.01
1-Nonanol	Alcohols	–	227.12±1.82	28.65±1.70	392.12±26.85	34.60±2.43	257.17±21.64	263.43±23.40	168.14±9.55	407.59±22.44	212.04±36.79
Nerol		3.37±0.40	22.29±2.49	10.02±1.93	50.39±2.37	26.28±1.83	7.04±0.41	5.38±0.17	63.57±12.33	12.00±1.06	7.65±0.94
Citronellol		7.83±0.73	281.51±7.30	23.01±4.33	307.01±51.98	184.84±21.57	5.63±0.51	5.09±0.66	53.09±5.73	5.02±0.29	166.89±7.77
Geraniol		27.18±5.14	4.78±0.94	20.82±1.29	5.74±0.65	20.38±1.96	9.33±1.65	1.48±0.26	6.40±0.48	48.51±7.90	80.40±0.34
1-Decanol		12.46±1.60	7.14±0.22	84.87±1.56	12.33±0.75	2.79±0.26	28.71±2.11	224.59±29.05	3.17±0.32	18.55±2.43	68.78±9.33
1-Dodecanol		14.91±1.93	11.78±0.62	5.44±1.15	117.30±12.25	3.77±0.20	6.03±1.04	11.09±1.35	63.22±3.17	1.61±0.25	59.47±3.58
Tetradecanol		18.72±0.98	62.59±6.74	221.21±26.68	61.59±4.10	136.82±20.85	33.06±7.00	20.86±1.06	24.84±3.14	19.81±3.38	28.76±3.59
Hexadecanol		111.46±8.92	5.34±0.23	4.79±0.76	11.18±1.95	83.31±14.44	140.41±10.71	5.72±0.31	64.91±10.69	22.90±1.93	142.57±3.35

Isophytol		–	170.66±14.17	11.09±0.42	97.63±2.47	156.86±19.70	56.36±1.41	4.44±0.62	117.30±13.89	31.07±6.56	191.98±24.65
Phytol		3.76±0.50	60.00±6.49	8.24±0.67	57.37±8.27	34.58±3.27	41.52±2.46	7.25±0.41	5.82±0.27	9.12±1.32	6.90±0.49
2-Methylpyrazine	Pyrazines	–	3.02±0.32	–	9.01±0.40	8.81±0.41	–	–	–	11.66±2.26	–
2,3-Dimethylpyrazine		–	–	–	35.80±1.15	–	–	–	–	–	–
2-Ethyl-3-methylpyrazine		–	–	–	–	–	–	–	–	41.07±3.21	–
Tetramethylpyrazine		–	–	–	66.43±10.02	–	–	–	–	–	–
Phenol	Phenols	18.21±0.67	2.95±0.34	1.08±0.12	16.63±2.01	19.35±0.64	0.93±0.06	28.91±3.13	8.43±1.02	1.13±0.10	1.42±0.12
4-Ethylguaiacol		–	–	–	–	–	–	–	–	–	1.01±0.04
4-Vinylguaiacol		–	–	1.78±0.18	4.70±0.61	–	18.18±2.58	0.95±0.03	–	–	–
Eugenol		0.42±0.03	27.27±3.81	0.90±0.11	1.78±0.15	37.88±5.67	60.73±6.86	2.42±0.28	3.54±0.59	2.37±0.29	9.15±0.90
Vanillin		–	0.77±0.08	1.92±0.27	1.29±0.16	0.70±0.03	1.66±0.30	1.05±0.06	3.01±0.17	44.45±3.61	14.06±2.37
2-Butylfuran	Furan	3.69±0.52	177.42±25.50	159.76±17.39	13.80±0.74	11.54±2.44	37.75±3.57	85.31±10.66	5.48±0.63	249.54±42.19	98.23±7.07
5-Methylfurfural		35.53±3.58	84.72±2.34	187.06±15.40	5.84±0.57	1.91±0.06	27.95±3.93	4.94±0.36	267.60±12.04	11.96±1.13	175.19±16.05
2-Pentylfuran		–	–	66.34±8.82	–	–	–	–	–	–	–
2-Hexylfuran		25.39±3.11	134.31±15.50	13.18±1.22	24.50±3.53	19.37±3.23	13.68±0.82	24.03±1.89	6.60±0.76	394.80±48.25	337.77±23.47
gamma-Butyrolactone	Lactones	–	–	–	–	2.18±0.17	–	–	46.55±2.77	64.11±8.43	1.86±0.22
gamma-Octalactone		0.76±0.07	75.23±8.69	5.17±0.91	5.42±0.70	21.93±1.92	3.93±0.32	26.77±2.80	3.51±0.50	13.10±1.66	20.42±0.37
delta-Octalactone		1.82±0.21	43.67±2.35	4.87±0.60	–	54.29±9.20	2.10±0.29	–	–	73.74±6.27	0.85±0.16
gamma-Decalact		0.99±0.17	1.41±0.15	13.40±1.40	3.20±0.07	5.87±0.83	0.93±0.02	3.85±0.47	0.96±0.18	17.41±1.69	3.45±0.13

one											
delta-Decalactone		–	98.04±4.24	19.30±1.96	1.80±0.11	29.82±0.46	4.90±0.29	2.38±0.24	1.11±0.06	98.05±12.15	8.11±1.51
delta-Dodecalactone		3.31±0.09	3.38±0.20	2.70±0.24	0.86±0.07	4.74±0.53	5.04±0.98	44.72±7.46	15.49±0.50	3.11±0.34	2.80±0.28
delta-Tetradecalactone		0.53±0.09	8.44±1.30	5.44±0.47	3.65±0.12	0.37±0.04	7.17±1.41	0.99±0.07	36.34±4.79	1.60±0.22	1.82±0.11
beta-Myrcene	Terpenes	–	–	–	–	–	1.75±0.29	–	–	–	–
D-Limonene		1.09±0.15	19.23±1.89	5.52±0.72	29.62±5.06	4.50±0.57	11.95±1.48	7.63±1.01	30.41±6.04	5.63±1.16	21.65±2.34
gamma-Terpinene		–	–	–	–	3.10±0.41	–	–	–	–	–
Caryophyllene		3.69±0.38	101.21±17.56	1.21±0.20	2.63±0.37	10.18±0.94	3.06±0.06	2.93±0.19	16.87±1.08	0.49±0.03	12.94±0.61
Nerolidol		0.58±0.10	5.31±0.50	14.37±2.51	73.70±9.52	0.53±0.03	1.12±0.17	2.72±0.32	2.44±0.35	3.07±0.27	1.28±0.12
Farnesol		–	6.99±0.66	–	19.99±1.07	1.81±0.22	42.55±3.47	–	3.24±0.24	–	–
2-Acetylpyrrole	Nitrogen-containing heterocyclic compounds	1.28±0.26	–	–	–	3.82±0.35	–	1.81±0.19	–	11.83±0.31	4.56±0.46
I–ole		–	2.10±0.08	53.80±10.91	40.99±0.96	1.91±0.21	22.98±4.72	16.46±0.76	1.61±0.25	26.45±0.52	2.12±0.10
Dimethyl disulfide	Sulfur-containing compounds	5.62±0.27	9.37±0.86	0.98±0.01	0.29±0.02	22.46±1.55	50.42±4.27	58.51±9.10	0.54±0.07	0.64±0.05	3.80±0.42
Methional		13.25±1.35	–	–	–	7.94±0.97	–	8.49±0.60	0.81±0.10	10.12±2.09	–
